# Spiniform phase-encoded metagratings entangling arbitrary rational-order orbital angular momentum

**DOI:** 10.1038/lsa.2017.156

**Published:** 2018-03-09

**Authors:** Kun Huang, Hong Liu, Sara Restuccia, Muhammad Q Mehmood, Sheng-Tao Mei, Daniel Giovannini, Aaron Danner, Miles J Padgett, Jing-Hua Teng, Cheng-Wei Qiu

**Affiliations:** 1 Department of Electrical and Computer Engineering, National University of Singapore, Singapore 117583, Singapore; 2 Institute of Materials Research and Engineering, Agency for Science, Technology and Research, Singapore 138634, Singapore; 3 Department of Optics and Optical Engineering, University of Science and Technology of China, Hefei 230026, China; 4 SUPA, School of Physics and Astronomy, University of Glasgow, Glasgow G128QQ, UK; 5 Information Technology University of the Punjab, Lahore 54000, Pakistan; 6 NUS Graduate School for Integrative Science and Engineering, National University of Singapore, Singapore 117456, Singapore; 7 The Edward S Rogers Department of Electrical and Computer Engineering, University of Toronto, Toronto M5S 3G4, Canada; 8 SZU-NUS Collaborative Innovation Center for Optoelectronic Science and Technology, Shenzhen University, Shenzhen 518060, China; 9 NUS Suzhou Research Institute (NUSRI), Suzhou 215123, China

**Keywords:** metasurface, nanophotonics, orbital angular momentum, superposition state

## Abstract

Quantum entanglements between integer-order and fractional-order orbital angular momentums (OAMs) have been previously discussed. However, the entangled nature of arbitrary rational-order OAM has long been considered a myth due to the absence of an effective strategy for generating arbitrary rational-order OAM beams. Therefore, we report a single metadevice comprising a bilaterally symmetric grating with an aperture, creating optical beams with dynamically controllable OAM values that are continuously varying over a rational range. Due to its encoded spiniform phase, this novel metagrating enables the production of an average OAM that can be increased without a theoretical limit by embracing distributed singularities, which differs significantly from the classic method of stacking phase singularities using fork gratings. This new method makes it possible to probe the unexplored niche of quantum entanglement between arbitrarily defined OAMs in light, which could lead to the complex manipulation of microparticles, high-dimensional quantum entanglement and optical communication. We show that quantum coincidence based on rational-order OAM-superposition states could give rise to low cross-talks between two different states that have no significant overlap in their spiral spectra. Additionally, future applications in quantum communication and optical micromanipulation may be found.

## Introduction

Light has many different properties that are described by its electromagnetic field. One of the most interesting properties of light is its ability to carry orbital angular momentum (OAM), which manifests itself as a helical wavefront with a phase singularity on the beam axis. Since its discovery in 1992^1^, the OAM of light has excited interest because it allows a new degree of freedom and a potentially unbounded number of quantum states for a light beam. The current commonly used technology has resulted in investigations using discrete integer OAMs for applications such as optical trapping and manipulation^2, 3, 4, 5, 6, 7, 8^, photon entanglement^9, 10, 11, 12^, astronomy^13^, microscopy^14, 15^, remote sensing and detection^16, 17^, optical communications^18, 19, 20^ and even integrated photonics^21, 22, 23, 24, 25, 26, 27, 28, 29, 30^. The rapidly developing exploitation of such diverse areas requires further development of OAM generation technology.

Hitherto, the devices for OAM generation have been primarily concerned with producing integer values of OAM states, even though one can theoretically continuously tune the OAM by changing the topological charges (TCs) of LG and Bessel beams^31, 32, 33^ or tailoring the ellipticity of Ince–Gaussian modes^34^. An OAM carrying beam has a helical phase *e*^*iℓφ*^ (where *ℓ* and *φ* are the winding numbers of the helical phase and angular coordinate, respectively)^1^, giving rise to an intensity annulus (i.e., doughnut) that is uniform for the integer *ℓ*, while for fractional *ℓ*, the intensity annulus is discontinuous with a phase step along *φ*=0. This smoothness leads to a similar influence on the design of the kinoform for generating the diffractive optical component, for example, fork gratings have smoothly varying fringes for integer *ℓ* and cutoff fringes with a discontinuity along *φ*=0 for fractional *ℓ*^35, 36^. This distinction makes it fundamentally difficult to transition between integer OAM and fractional OAM in a static device, resulting in poor reconfigurability since different OAM states must be individually addressed by separate devices or phase profiles^21, 22, 23, 37, 38, 39, 40^. Digital devices such as spatial light modulators (SLMs)^41^ and digital micromirror devices (DMDs)^42^ have been used to generate different OAM values. However, their pixel resolution limits lead to spatial phase jumps and account for inaccuracies of fractional OAM (see Section 1 in Supplementary Materials). Therefore, the community has to explore the applications of such digital devices (such as those for quantum entanglement) on the basis of the integer or fractional-order OAM^9, 10, 11, 12, 43, 44^.

Furthermore, tunable or continuous OAMs have recently received increasing attention for applications like path-OAM-interfaced quantum entanglement^45^ and optical successive micromanipulation^46^. Attempts have been made to generate tunable OAMs using indirect methods such, as the weighted superposition of two cross-polarized beams^46^, the interference of two vortices^47^, internal conical diffraction^48^ and optical geometric transformations^45, 49, 50, 51^. Although these methods offer a new degree of control for the OAM of light, they are intrinsically accompanied by either poor beam quality, very limited tunable ranges or complicated transformations that require optical correction after long-distance propagation. Novel approaches are highly desired for exploring and extending the applications of OAMs in a rational-order manner.

Here, we report a continuous OAM transmitter including bilaterally symmetric gratings with an aperture that produces arbitrary rational-order vortex beams carrying OAMs without any theoretical limit. Distinguished from other vortex beams (e.g., LG and Bessel beams) that change their OAMs by changing TCs, our rational-order OAM beam has a spiniform wavefront with phase singularities located equidistant along a line and tunes its average OAM by changing the number of singularities that the beam accommodates. This approach realizes both non-integer and arbitrary rational-order generation of OAM across the full range by transmitting these phase singularities through the aperture and enables the exploration of quantum entanglement based on such continuous OAMs for communication purposes.

## Materials and methods

**Figure 1:**
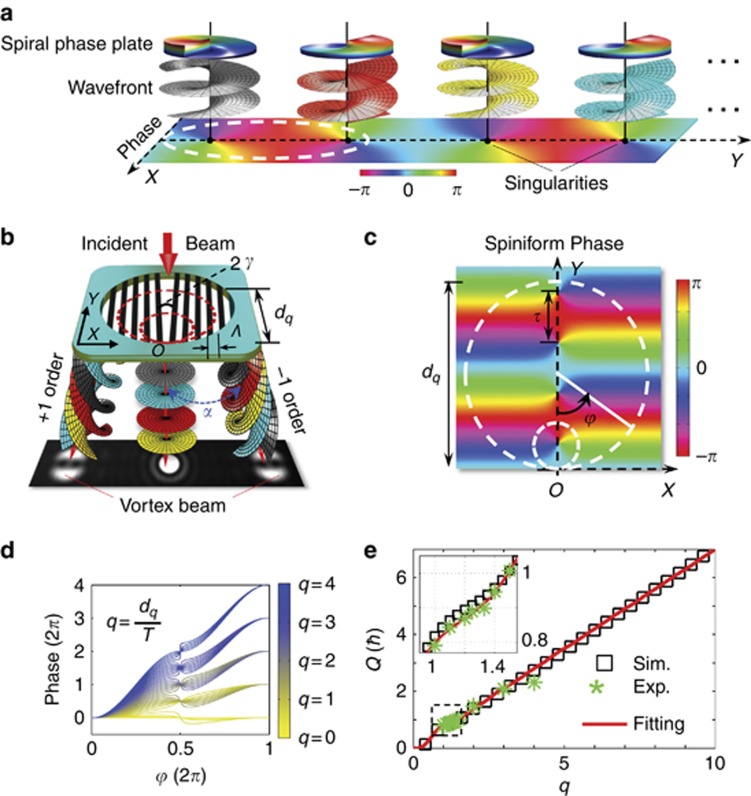
Mechanism of the analog vortex transmitter. (**a**) Light obtains a helical wavefront with spatially separated phase singularities (black dots) by passing through four transversely located SPPs. (**b**) Sketch of the transmitter composed of two inclined (inclination angle of *γ*) gratings with a period of *Λ* at both sides and a circular aperture of varying diameter (*d*_*q*_), which geometrically acts as an excircle (red dashed circles) tangent to the *x*-axis at a reference point *O*. (**c**) Phase profile encoded into the vortex transmitter. *τ* denotes the spatial distance between two neighboring phase singularities. *φ* is the angle coordinate of the circular aperture and increases anticlockwise from *φ*=0 (negative *y*-axis) to 2*π*. (**d**) Phase along the circumference (dashed circle in (**d**)) of the circular aperture for its corresponding *q*. The phase at *φ*>*π* is unwrapped by adding 2*π*. The curves denote the phase values for *q* (distinguished by the curve colors). (**e**) The average OAM (*Qћ*) of a photon as a function of *q*. The fitting curve (solid red line) of the simulated results (black square boxes) exhibits a root mean square error of 0.04, while the experimental results are denoted by greenish asterisks. Inset: Zoom-in of the data between *q*=1 and *q*=1.5.

Traditionally, light with a planar wavefront can increase its OAM by successively passing through *ℓ* concentric and vertically located spiral phase plates (SPPs), each of which has a TC of 1^35^. Similarly, light could also obtain an OAM by passing through a series of transversely located SPPs (Figure 1a), which have wavefronts with spatially separated singularities. One can increase the OAM of light by including more SPPs, leading to more phase singularities in the wavefront of the light. Hence, when a phase profile with regularly distributed (e.g., periodic) singularities is encoded into a beam generator, we infer that the optical vortices will be smoothly emitted, making it possible to generate a continuous OAM by employing a gradually varying aperture.

To realize this, we propose using a bilaterally symmetric metagrating with an aperture as a vortex transmitter, whose working principle is sketched in Figure 1b. With its *y*-axis at the line of symmetry, this transmitter consists of two gratings with a tilting angle *γ*. A circular aperture is placed above the metagratings, and its diameter *d*_*q*_ can vary along the *y*-axis. For a normally incident plane wave, the transmission function of this transmitter can be expressed as


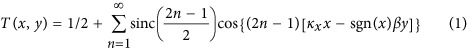


where sinc(*x*)=sin(*πx*)/(*πx*), the diffraction order *n* is a positive integer, *κ*_*x*_ is a constant determining the diffraction angle, *β* stands for a constant phase gradient along *y* direction, and sgn(*x*) refers to the sign function of the variable *x* (and is mathematically responsible for the bilateral symmetry of the structure). The metagrating parameters, such as the period *Λ*=2*π*/(*κ*_*x*_^2^+*β*^2^)^1/2^ and the inclination angle *γ*=tan^−1^(*β*/*κ*_*x*_), are derived in the Supplementary Materials.

Light from the first-order diffraction (i.e., *n*=1) possesses a linearly *y*-dependent phase function:


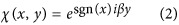


where sgn(*x*) accounts for the opposite phase variation tendency, such that *χ* increases for positive values of *x* and decreases for negative values of *x*. To determine the phase singularities, we show the phase profile after a low-pass filter (see Section 2 in the Supplementary Materials) in Figure 1c, removing the phase jump along the *y*-axis. Due to its linear *y* dependence, a phase difference between both sides occurs periodically along the interface, leading to phase singularities at equal spacings of the spatial interval *τ*. Within one cycle of the 2π phase, the number of phase jumps reaches its maximum of π at a phase singularity twice, which means that the phase difference spanning a distance of *τ* along *y* is *βτ*=*π.*

Acting as a regulator, the aperture smoothly changes its diameter along the *y*-axis of symmetry to precisely control the linear output of phase. To quantify this output, we introduce a dimensionless parameter: the singularity strength *q*≡*d*_*q*_/*τ*. Because the aperture size *d*_*q*_ can be smoothly tuned, *q* smoothly varies its integral and fractional values to realize the continuous generation of optical vortices by a single transmitter. We plot the phase along the circumference of the aperture for different *q* values in Figure 1d, showing a phase change of 2π[*q*], where[*q*] denotes the round of *q* and is equal to the number of encircled phase singularities. As expected, our results in Figure 1e reveal that this vortex beam has an average OAM of *Qћ* (*ћ* is the reduced Planck constant) for a photon with


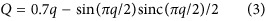


which will be discussed in detail later.

## Results and discussion

Considering the operating wavelength (*λ*<*Λ*) and fabrication issues, we experimentally applied the following specifications to the sample fabrication: *Λ*=1 μm, *γ*=tan^−1^ (1/240) and, correspondingly, *τ*=120 μm. This transmitter was patterned on a 100-nm thick chromium film deposited on a quartz substrate via electron beam lithography and a dry etching process. To achieve high-fidelity experimental results, the apertures were directly fabricated on transmitters, leaving the individual samples with different *q* values. Two groups of specimens with integer *q*=1–4 and fraction *q*=1.1–1.5 values were fabricated to exemplify the analog generation concept of rational OAMs. The scanning electron microscopy (SEM) images of the fabricated samples are provided in Section 3 of the Supplementary Materials.

**Figure 2:**
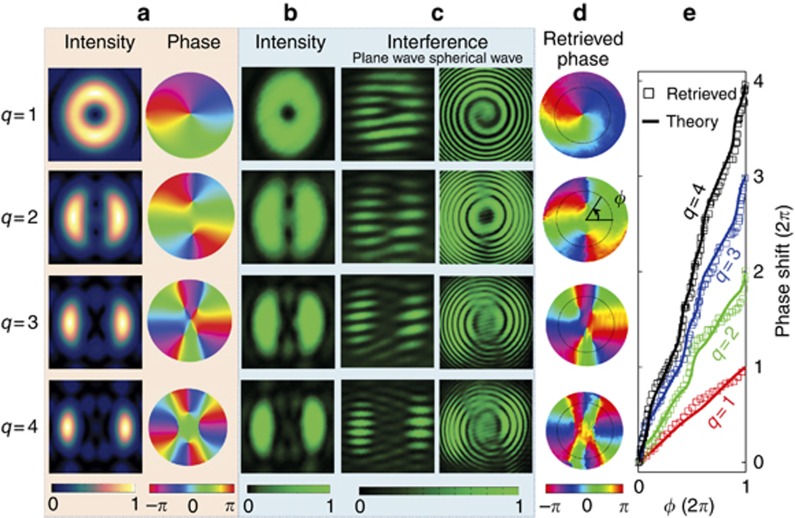
Optical vortices with integer *q* values. (**a**) Simulated intensity and phase profiles for *q*=1, 2, 3 and 4, whose *Q*=0.798, 1.4, 2.126 and 2.8, respectively. (**b**) Measured intensity profiles of the far field. (**c**) Experimental interference patterns with plane (left) and spherical (right) waves. (**d**) Phase profiles retrieved from experimental interference patterns with planar waves. *Φ* denotes the angular coordinate. (**e**) Quantitative comparison of the azimuthal phase shift (Δ*P*≡*P*(*Φ*)−*P*(*Φ*=0), where *P* is the phase of this vortex beam) between the experiment (curves) and simulation (square boxes). Data are obtained along the black dashed circles as shown in **d**.

Figure 2 shows the simulated and experimental results of the integer group (*q*=1–4) at a wavelength of 532 nm. Under the assumption of uniform illumination, the simulated intensity and phase profiles of the light from the first-order diffraction in the Fraunhofer region are shown in Figure 2a. At *q*=1, an elliptical transverse profile is formed with a single phase singularity, which splits into a two-lobed shape from *q*=2 onwards due to the spatial mismatch of the singularities. As *q* increases, the central darkness expands to accommodate more phase singularities, moving the two lobes farther apart. Meanwhile, these two lobes shrink due to gradually weakening diffractions when the aperture continues to increase^52^. The simulated intensity profiles are well validated by the measurements in Figure 2b. Such an intensity profile originates from the interactions between the spiniform phase and operating circular aperture during its paraxial propagation, which act as low-pass filters in the Fraunhofer region^52^; see Section 4 in the Supplementary Materials.

The optical wavefronts were experimentally revealed in Figure 2c by the interference with a reference Gaussian beam via a Mach–Zehnder interferometer. The dislocated fringes of the plane-wave case and the spiral arms of the spherical-wave case have been revealed in the interferograms. The respective TC is quantifiable through the number of dislocated fringes for the plane-wave case or through the number of arms for the spherical-wave case. Note that the simulated interference patterns (see Sections 5 and 6 in the Supplementary Materials) agree very well with the measured results. Figure 2d plots the corresponding phase profiles retrieved from the experimental results of the plane-wave interference using the Fourier transformation^53^. The retrieval was validated by the simulation, exhibiting nearly identical phase distributions in Figure 2e. The phase shift accumulated along a closed circle is 2*πq*, which quantifies the integer TC of *q*.

**Figure 3:**
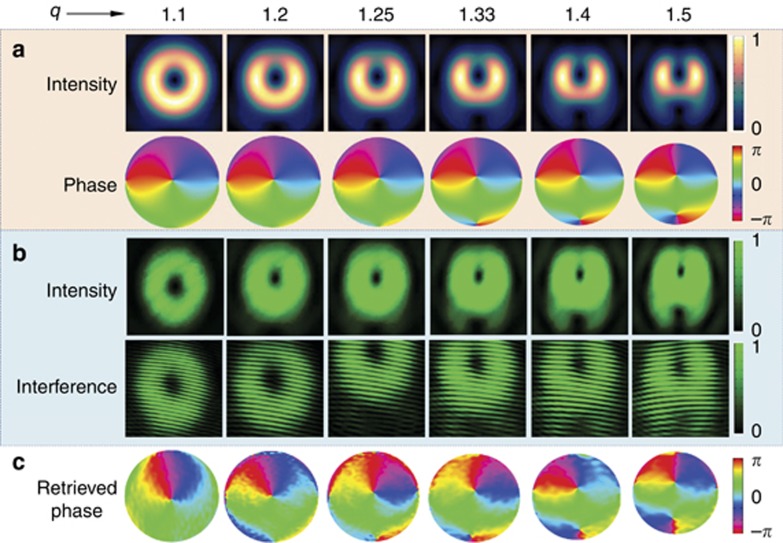
Optical vortices with fraction *q* values. (**a**) Simulated intensity and phase profiles for *q*=1.1, 1.2, 1.25, 1.33, 1.4 and 1.5, whose *Q*=0.839, 0.868, 0.884, 0.913, 0.946 and 1.007, respectively. (**b**) Measured intensity profiles (upper) for the different *q* values and their corresponding interference patterns (lower) with planar waves. (**c**) Phase profiles reconstructed from experimental interferences with planar waves.

The fractional group (*q*=1.1–1.5) has been examined under the same conditions, and their results are shown in Figure 3. As *q* increases, the predicted transverse intensity profile in Figure 3a evolves from an ellipse to an H-shape, and its bottom half tends to enclose a dark core of phase singularity. This result agrees with the measured intensities in Figure 3b, including the experimental interference patterns. The dislocated fringes are enhanced with increasing *q*, which is attributed to the fact that the neighboring singularity is gradually dominated. The retrieved phase profiles (Figure 3c) agree well with the simulated results (Figure 3a). Additionally, an animation of the continuous generation of these optical vortices with varying *q* can be found in the Supplementary Movie.

To show the connection between our vortex beam and LG beams, we decompose the spiniform phase in Equation (2) in terms of the angular-dependent helical phase


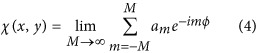


where the coefficient


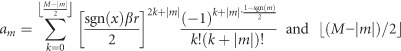


denotes the integer part of (*M*-|*m*|)/2. Equation (4) implies that our vortex beam can be written as a weighted superposition of spiral modes and possesses the average OAM per photon, which is similar to the Ince–Gaussian modes^33^ and fractional-order LG modes^36^.

To quantify its analog effect, the average OAM carried by this vortex beam has been investigated theoretically (see Section 7 in Supplementary Materials) and experimentally in Figure 1e. The experimental amplitude and phase profiles of our vortex beam in the far field could be obtained with the phase retrieval method, as sketched in Supplementary Fig. S7 of the Supplementary Materials. The average OAM (*Q* in units of *ћ*) per photon of the vortex beam is evaluated by using Supplementary Equation (S17) of the Supplementary Materials and is finally correlated as a function of *q* via Equation (3), which is a fit of the simulated results. Due to the coupling of the sine and sinc functions, this result shows nonlinearity within the interval of [0, +2], beyond which quasilinearity governs the relation between *Q* and *q* in the rational range. This is distinct from the pure nonlinear relationship of the LG and Bessel beams^31, 32, 33^. Thus, the average OAM of such a novel vortex beam has been validated as continuously addressable in rational states without any theoretical limit.

### Quantum spiral spectrum

In spontaneous parametric downconversion, OAM-entangled photon pairs have the quantum state^54, 55^


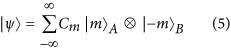


where *C*_*m*_ is the probability amplitude of finding one photon in the signal mode 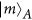 and one photon in the idler mode 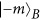

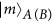
 indicates the optical mode that has one photon with a quantized OAM of *mħ* in the signal (idler) arm and 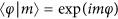.

**Figure 4:**
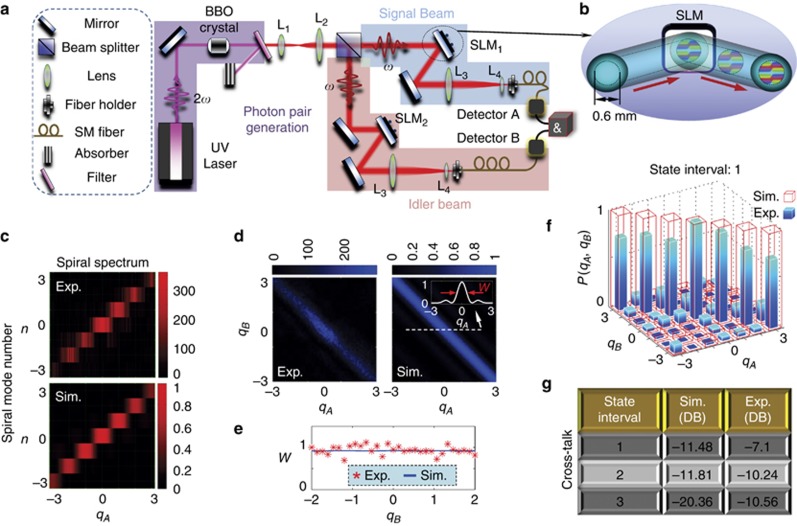
Quantum operation. (**a**) The experimental setup based on spontaneous parametric downconversion (SPDC). (**b**) Modulating light with the required phase profiles in a reflective SLM. (**c**) Quantum spiral spectrum of the generated vortex beam. (**d**) Quantum coincidences between a vortex beam with *q*_*A*_ in the signal beam and that with *q*_*B*_ in the idler beam. Inset: Line-scan simulated coincidence as a function of *q*_*A*_ when *q*_*B*_=0. (**e**) The experimental and simulated width *w* as a function of *q*_*B*_. (**f**) Quantum coincidences between our vortex beams with discrete *q*_*A*_ and *q*_*B*_ (=0, ±1, ±2, ±3). (**g**) The cross-talk of the quantum coincidences for the different state intervals of 1, 2 and 3.

Since our fabricated vortex transmitter has a largest diameter of 480 μm, it is quite challenging to select our vortex beam by using an additional aperture. Thus, the signal beam in the experimental setup given in Figure 4a is imaged on SLM_1_ is imparted with the spiniform phase (see Figure 4b) to facilitate achieving our fractional OAMs. Note that the vortex beams generated by the spiniform phase-encoded SLM (see Section 8 and Supplementary Fig. S8 of Supplementary Materials) is completely identical to those created by the above vortex transmitters. The only difference is that the SLM cannot, in principle, generate a rigorously continuous OAM. However, this difference will not change the intensity and phase profiles of the proposed vortex beams and, therefore, is still valid for verifying the feasibilities of the use of our vortex beams for quantum operation.

The spiniform phase-encoded SLM will enable the selection of an OAM-superposition state 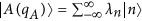, where 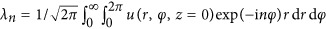; the spiral spectrum is *γ*_*n*_=|*λ*_*n*_|^2^/*T*; and *T* is a normalization factor, such that 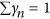 (Ref. 56). Similarly, the idler beam modulated by SLM_2_ is imparted with a helical phase for generating an OAM eigenstate 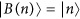. Both resulting beams are separately imaged at the facets of single-mode fibers and are then coupled to avalanche photodiodes for detection. The photodiodes are connected to a coincidence circuit that will allow the recording of the coincidence rate as a function of the states specified by the SLM, thus, by scanning the OAM eigenstate in the idler beam. Thus, one can obtain the coincidence probability


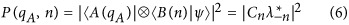


where the superscript ‘*’ indicates the complex conjugate. Equation (6) can also be taken as the quantum spiral spectrum due to the existence of *C*_*n*_(Ref. 57). For a maximum entanglement^55^, *C*_*n*_ is taken as a constant for all the simulations in this paper.

Figure 4c shows the measured and simulated quantum spiral spectra with good agreements. To decrease the experimental error caused by the limited photon flux^58, 59, 60^, the measured spiral spectrum is evaluated by calculating the quantum contrast for each coincidence measurement, which allows us to express our results as a function of the strength of the quantum correlation. The quantum contrast is defined as the ratio of the recorded coincidence rate to the expected accidental coincidence rate, where the accidental coincidences are calculated by multiplying the time resolution (refer to Ref. 59) of our coincidence counting electronics with the count rates detected by detectors A and B (see Figure 4a)^59, 60^. In Figure 4c, the experimental quantum contrast gets smaller at larger |*q*_*A*_| values, which is mainly attributed to the limited quantum spiral bandwidth of the system^58^ and the increasing noise. As *q*_*A*_ changes in our experiment, the smooth spiral spectrum confirms that the proposed mechanism is valid for manipulating the OAM at the single-photon level.

### Quantum coincidence

Quantum coincidence is carried out by generating two vortex beams with *q*_A_ and *q*_B_ in the signal and idler arms. The vortex beam in the idler arm has an OAM-superposition state 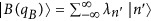. The coincidence rate, as a function of *q*_*A*_ and *q*_*B*_, can be obtained by


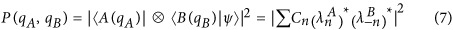


The experimental coincidence per 4 s is provided in Figure 4d, which is consistent with the simulation results. The diagonal elements with *q*_*A*_=−*q*_*B*_ are nearly uniform for the maximum values from both the simulations and experiments. These results indicate that the total angular momentum is also conserved in the spontaneous parametric downconversion process for the OAM-superposition states, which behaves like the case of the OAM eigenstates^9^.

The coincidence rates decrease gradually when both the *q*_*A*_ and *q*_*B*_ parameters deviate from *q*_*A*_=−*q*_*B*_. To incorporate this effect, a line-scan simulated coincidence at *q*_*B*_=0 is shown with a width of *w* (which is evaluated by the full-width at its half-maximum) in the inset of Figure 4d. For a given *q*_*B*_, the width *w* determines the range of *q*_*A*_ where the coincidence is high. The simulated and experimental widths as functions of *q*_*B*_ are located at ∼0.925, see Figure 4e. The significance of this result is twofold. First, the vortex beams with discrete *q* values are preferred to avoid the strong cross-talks between two neighboring states. Second, the state interval (i.e., the minimum difference in OAMs between two states) should be larger than 0.925 to decrease the cross-talk.

Figure 4f shows the simulated and experimental coincidences between these discrete states (*q*_*A*,*B*_=0, ±1, ±2, ±3) with intervals of 1. Similarly, the maximum coincidence occurs when the diagonal elements obey *q*_*A*_=−*q*_*B*_, as confirmed in both the simulated and experimental results. The coincidence rate of the non-diagonal case stands for the noise and should be suppressed to achieve a low cross-talk. The maximum probability among these non-diagonal cases is 0.0711 (the cross-talk is 10log_10_(0.0711)=−11.48 dB) in the simulations and 0.1952 (indicating a cross-talk of −7.1 dB) in the experiments. This discrepancy mainly originates from the imperfect generation of our vortex beam caused by SLM pixilation (i.e., the pixel pitch of 15 μm in our SLMs) and the small aperture (0.6 mm in diameter) of the efficient phase of the SLM, which leads to increased noise due to the decreased photon flux used for detection (see Section 8 in the Supplementary Materials). When the state interval is greater, the cross-talk could be further suppressed due to the overlapping of the spiral spectra between two neighboring states becoming smaller. Figure 4g shows that the experimental cross-talks are −10.24 dB for the interval 2 (with *q*_*A*,*B*_=±1, ±3) and −10.56 dB for the interval 3 (with *q*_*A*,*B*_=0, ±3), which are comparable to the pure-OAM-based communication requirements^18, 19, 61^. The experimental and simulated results for intervals 2 and 3 are provided in Section 8 and Supplementary Fig. S9 of the Supplementary Materials.

From the simulated and experimental results, one can find that our vortex beam is able to select the superposition states of OAMs for quantum operations, although this selection is realized by using a phase-type SLM. We have to emphasize that a rigorously continuous generation of rational OAM must refer to the proposed mechanism of our metagratings combined with a smoothly tunable aperture. We also note that two issues should be addressed when carrying out quantum operations using continuous OAMs. First, the total size of the metagratings should be large so that a tunable aperture is available in practice. In this work, the largest diameter of our metagratings is ~480 μm, which is too small for a commonly used aperture. The fabrication of large-scale metagratings can be achieved by using laser direct-writing techniques. Second, the pump laser in the spontaneous parametric downconversion process should be strong enough to enhance the signal-to-noise ratio of the quantum coincidence because the total efficiency of our binary-amplitude gratings has a theoretical value of ~10%.

## Conclusions

We have rigorously demonstrated the concept of continuous OAM. The generating optical element is based on periodic gratings of bilateral symmetry with tunable apertures. In addition, the mechanism tailoring the OAM of light via the number of involved phase singularities provides unique insights for investigating the superposition states of OAMs in quantum physics and singular optics. We have demonstrated the feasibility of realizing quantum coincidence by using the OAM-superposition state, which might benefit quantum physics and technology^62, 63, 64^. Arbitrarily maneuvering OAM across rational states makes is an attractive method for enriching electron vortex beams^65^, spiral imaging techniques^56, 57^ and optical continuous manipulation for the effective sorting or selection of microparticles^66^.
