# Supplementary material for: Spiniform phase-encoded metagratings entangling arbitrary rational-order orbital angular momentum
Source: Light Sci Appl. 2018 Mar 9;7:17156–. doi: 10.1038/lsa.2017.156 (PMC6060047; doi:10.1038/lsa.2017.156)
Supplement: Supplementary Materials [file lsa2017156x1.pdf]

Supplementary Materials

Spiniform phase-encoded metagratings entangling arbitrary rational-order orbital angular momentum

Kun Huang[1,2,3*], Hong Liu[2*], Sara Restuccia[4*], Muhammad Q. Mehmood[5], Shengtao Mei[1,6], Daniel Giovannini[7], Aaron Danner[1], Miles J. Padgett[4], Jinghua Teng[2†], and Cheng-Wei Qiu[1,6,8,9†]

[1] Department of Electrical and Computer Engineering, National University of Singapore, 4 Engineering Drive 3, Singapore 117576, Singapore

[2] Institute of Materials Research and Engineering, Agency for Science, Technology and Research, 2 Fusionopolis Way, Innovis, #08-03, Singapore 138634, Singapore

[3] Department of Optics and Optical Engineering, University of Science and Technology of China, Hefei, Anhui 230026, China

[4]SUPA, School of Physics and Astronomy, University of Glasgow, Glasgow G128QQ, UK

[5] Information Technology University of the Punjab, Lahore 54000, Pakistan

[6]NUS Graduate School for Integrative Science and Engineering, National University of Singapore, Singapore 117456, Singapore

[7] The Edward S. Rogers Department of Electrical and Computer Engineering, University of Toronto, Ontario M5S 3G4, Canada

[8]SZU-NUS Collaborative Innovation Center for Optoelectronic Science and Technology, Shenzhen University, Shenzhen 518060, People's Republic of China

[9]NUS Suzhou Research Institute (NUSRI), Suzhou Industrial Park, Suzhou 215123, People's Republic of China

* These authors contributed equally to this work

[†]Correspondence and requests for materials should be addressed to C.W. Q. (email: eleqc@nus.edu.sg) or J. H. T. (email: jh-teng@imre.a-star.edu.sg).

Section 1. Inaccurate demonstration of fractional OAM for digital devices (i.e., SLM and DMD)

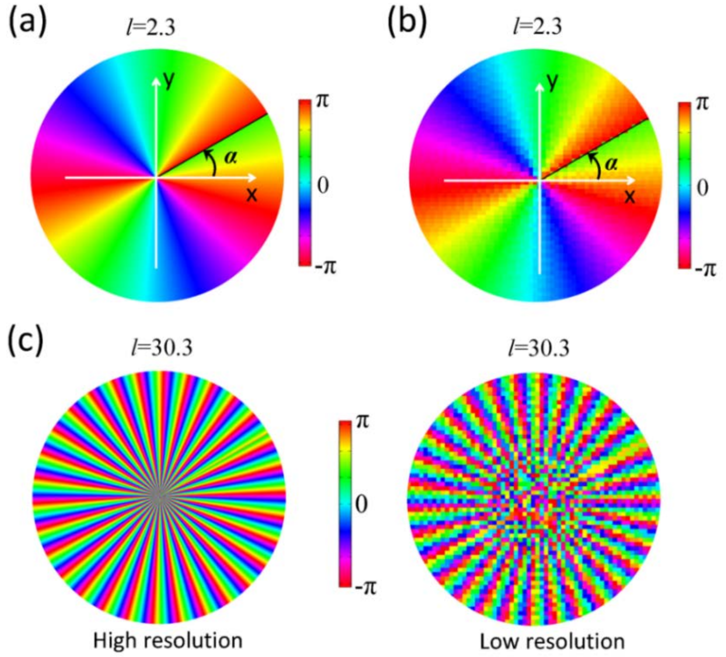

Figure S1. Phase profiles of a fractional OAM with high and low resolution. For low-resolution case, the pixel pitch has the size of 6.4μm*6.4μm, which is the smallest one in industry. (a-b) *l*=2.3 with high and low resolution. (c) *l*=30.3 with high and low resolution.

For a helical phase $e^{i\ell\varphi}$, the phase profile along the angle direction is continuous for integer *l* and continuous for fractional *l* because of phase jump at φ=0. More generally, the position of phase jump is located at φ=α (black line in Fig. S1(a)), which is an important parameter in implementing quantum entanglement in terms of fractional OAMs [S1]. When one uses the digital devices such as spatial light modulator and digital micro-mirror device to demonstrate the helical phase with fractional *l*, their fixed pixel pitches will fail to demonstrate an inaccurate straight line, as shown in Fig. S1(b). Although this inaccurate demonstration of digital devices is usually ignored for small *l*, this inaccuracy will be quite significant for large *l* as shown in Fig. S1 so that it leads to a clear reduction of mode transformation efficiency in quantum entanglement [S2]. Therefore, the digital SLM and DMD are not perfect in some special applications such as high-quanta quantum entanglement in terms of orbital angular momentum and optical manipulation.

Section 2. Bilateral Symmetrical Structure

The structure mathematically described by Eq. (1) in the main text has a bilaterally symmetric pattern as shown in Fig. S2(a). Such a structure can provide a perfect resolution in generating an optical vortex beam because it is not pixelated as SLM and DMD. We can obtain the basic information (i.e., grating period Λ and inclination angle γ) of this vortex transmitter in Eq. (1), where the item related to the spatial coordinates of *x* and *y* is

$$f(x,y) = \kappa_x x - \mathrm{sgn}(x)\beta y\,, \tag{S1}$$

which is responsible for the structural parameters (i.e., grating period Λ and inclination angle γ) of the transmitter. To unveil this, Eq. (S1) can be further modified as

$$f(x, y) = \sqrt{\kappa_x^2 + \beta^2}\left[\cos(\gamma)x - \mathrm{sgn}(x)\sin(\gamma)y\right], \quad \text{(S2)}$$

where $\cos(\gamma) = \kappa_x \big/ \sqrt{\kappa_x^2 + \beta^2}$ and $\sin(\gamma) = \beta \big/ \sqrt{\kappa_x^2 + \beta^2}$, leading to $\tan\gamma = \beta/\kappa_x$. Considering that Eq. (1) has the form of cos[*f*(*x*,*y*)], we can directly get the grating period $\Lambda = 2\pi \big/ \sqrt{\kappa_x^2 + \beta^2}$. Thus, we have the constant β and κ (in terms of Λ and γ)

$$\beta = \frac{2\pi \tan\gamma}{\Lambda\sqrt{1+\tan^2\gamma}}, \quad \text{(S3)}$$

$$\kappa_x = \frac{2\pi}{\Lambda\sqrt{1+\tan^2\gamma}}. \quad \text{(S4)}$$

This means that constant β depends on the grating period Λ and its inclination angle γ. Once the grating is fixed, one can get the parameters β and κ_x, which respectively determines the distance between two phase singularities and its broadband behaviour that will be discussed in details later.

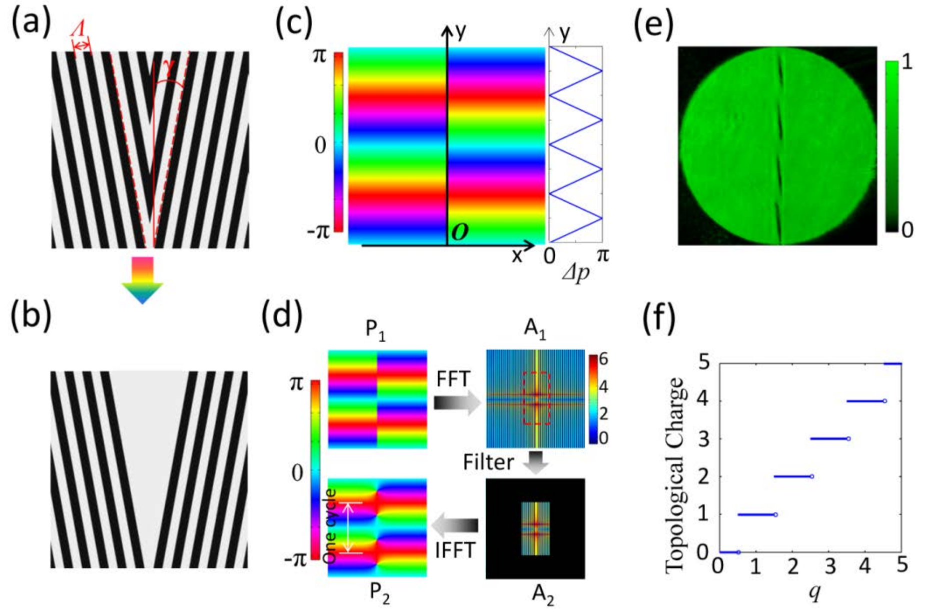

Figure S2. Vortex transmitter and its encoded phase. (**a**) Sketch of vortex transmitter directly described by Eq. (1) in the main text. The white and black bars represent transparent and opaque areas of the transmitter. The triangles in between two dashed red lines are removed to form the proposed transmitter, as sketched in (b). (**c**) The encoded phase profile *P*(*x*,*y*) directly described by Eq. (2) in the main text. A clear phase jump from positive *x* to negative *x* happens at the y axis. Inset: Phase difference ΔP along *y* between negative x and positive x. $\Delta P = P(x,y) - P(-x,y)$. (**d**) The low-pass filtering operation for unveiling the phase singularities. $P_1 = \arg\langle\chi(x,y\rangle$ is the phase profile in (c). $A_1 = \log_{10}|\mathrm{FFT}(P_1)|$ denotes the frequency spectrum and the logarithm operation is employed for a better show of frequency. A_2 is the low-passed frequency spectrum obtained by selecting the spectrum in the dashed red rectangle and padding zero (black) in other regions. $P_2 = \mathrm{IFFT}(A_2)$ shows the obvious phase singularities. (**e**) Experimental intensity profile captured by an objective lens (10X, 0.3 NA) slightly above the exit plane of transmitter with *q*=4. The four dark regions mean four phase singularities. (**f**) Relationship between topological charge of phase enclosed in a circular aperture and its corresponding *q*. It shows only integer topological charge is obtained.

In order to decrease the technical requirement in fabrication, we remove all the structures between two dashed lines in Fig. S2(a) and make it transparent in our practical vortex transmitter shown in Fig. S2(b). Thus, the acute vertexes along y axis will not appear in our transmitter, which will significantly decrease the fabrication difficulty when the structure is scaled down to a small size. Interestingly, the generated vortex beam at the far field is nearly the same as that generated by structure in Fig. S2(a). This originates from two aspects: 1) the far-field performance of our transmitter is dominated by two bilaterally symmetrical gratings that bring the inversed phase, leading to a dislocation; 2) light at the far field is usually not influenced by the slight defect at the near field. It can refer to the simulated and measured vortex beams (Fig. 2 in main text) that behave consistently with each other as expected.

The encoded phase profile described by Eq. (2) of main text is shown in Fig. S2(c), in which there is a clear phase jump from negative x to positive x. The phase difference between both sides is plotted in the inset of Fig. S2(c), showing a periodic change with the largest value of π. The phase singularities exist at the positions with phase difference of π. For both sides of y axis, the changing speed of phase is determined by the gradient β. According to Fourier optics [S3], this phase jump always implies a high spatial frequency in frequency domain, which cannot be maintained at the propagation of light that can be taken as a low-pass filter in free space. In this case, we implement a low-pass filtering of phase profile of Fig. S2(c) to remove this phase jump and unveil the hidden phase singularities. The operation details are shown in Fig. S2(d). Apparently, the filtered phase has a smooth change around the y axis while keeps the phase unchanged in other regions, leading to a spinform vortex phase with two singularities in one cycle. This implies a simple result between τ and β: $\tau\beta=\pi$. Then we have $\tau=[\Lambda\cdot(1+\tan^2\gamma)^{1/2}]/(2\tan\gamma)$, which shows a straightforward relationship between the phase singularities and the structural parameter (Λ and γ) of transmitter.

These enclosed phase singularities can be experimentally manifested by the zero-intensity (dark) regions in intensity profiles of Fig. S2(e), captured slightly above the exit plane of vortex transmitter for the exemplary case q=4. Because the phase difference between both sides is periodically formed along y aixs as shown by the inset of Fig. S2(c), the dark regions around phase singularities in the captured intensity profiles extends vertically and forms several dark slits with equal interval. Due to Guoy phase shift [S4], these dark slits are a little tilting because the intensity is captured slightly above the exit plane.

We show the relationship between the topological charge and q in Fig. S2(f). Its topological charge is valued at integer [q], which is distinguished from Laguerre-Gaussian and Bessel beams with a helical phase ($e^{im\varphi}$) that are able to have fractional topological charge. This can be interpreted by the smooth of our spiniform phase, which obeys the definition of topological charge by M. V. Berry. However, with the increment of q, the jump of its topological charge between integers will not impose an influence on the smooth generation of its carried OAM that is tightly dependent on both intensity and phase of vortex beam, which has been clearly shown in Fig. 1(e) and Eq. (3) of main text.

Section 3. Fabrication of vortex transmitter

In order to show the high-fidelity experimental result, we fabricated the apertures directly on the vortex transmitter with their vertical distance of zero, so that the error from light diffraction between them can be eliminated. But, this will not affect the concept of analog generation because the double grating in the transmitter is fixed and only the aperture is changed. In fact, when the grating period is large (e.g. up to 20μm), the whole size of transmitter will increase correspondingly up to several millimetres, allowing the traditional iris aperture to realize the smooth output of optical vortices.

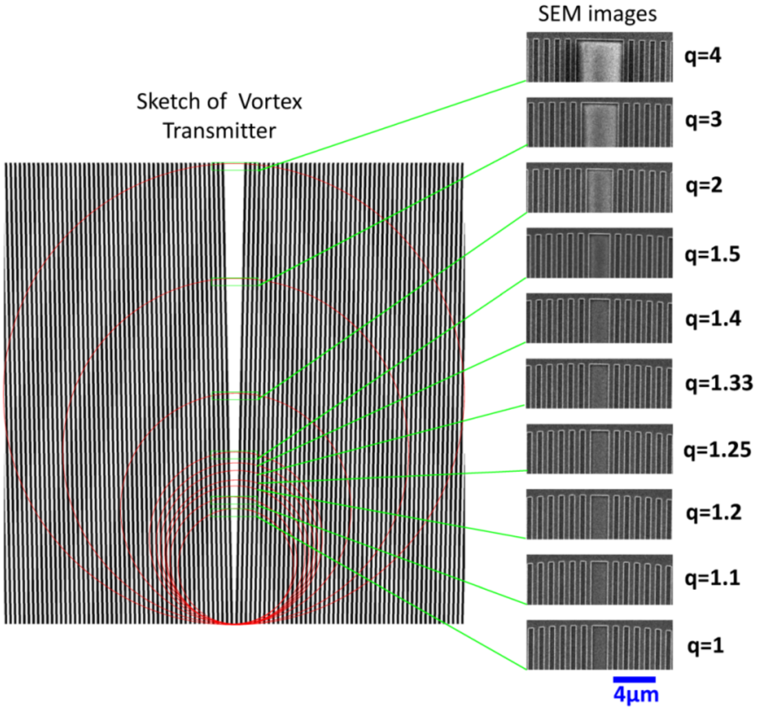

Figure S3. Fabricated samples of vortex transmitter. The apertures denoted by the red circles are directly fabricated on the transmitter, leaving individual samples whose SEM images with 3000X magnification are provided in right volume. Correspondingly, the integer group has individual samples of q=2 (d2=240 μm), q=3 (d3=360 μm) and q=4 (d4=480 μm) while the fractional group consists of q=1.1 ($d_{1.1}$=132 μm), q=1.2 ($d_{1.2}$=144 μm), q=1.25 ($d_{1.25}$=150 μm), q=1.33 ($d_{1.33}$=159.6 μm), q=1.4 ($d_{1.4}$=168 μm) and q=1.5 ($d_{1.5}$=180 μm), respectively. The scale bar is available for all SEM images.

The SEM images of fabricated samples are shown in Fig. S3. To better exhibit the details, we only provide the top parts of SEM images for all the fabricated samples. Because the aperture increases, their top parts widen gradually, as shown by their images. The fabrication processes are shown below:

The binary mask was fabricated on 100 nm thick chromium (Cr) film which was deposited by electron beam evaporator (Denton Vacuum, Explorer) at room temperature under a vacuum pressure of 5 x 10^{-7} Torr on top of a quartz substrate. ZEP-520A resist mixed with anisole (1:1 ratio) was spin-coated over Cr at a speed of 5000 rpm and then soft-baked at 180°C on a hotplate for a period of 120s. After that, it underwent electron beam lithography (ELS-7000, Elionix) to expose the specimen at a dosage of 240 $\mu C/cm^2$, beam current of 50 pA and acceleration voltage of 100 kV followed by development in o-Xylene for a period of 30s. The specimen was then subjected to a dry etching process (Nanoquest, Intlvac) under conditions of beam voltage/current of 300 V/110 mA, acceleration voltage/current of 100 V/6 mA, power of 180 W and vacuum pressure of about 2.54 x 10^{-4} Torr for approximately 7 minutes. After that, the residual resist was removed by soaking the specimen in Microposit™ remover 1165 (Shipley) for 12 hours followed by oxygen plasma etching (Oxford RIE) under a power of 100 W and O_2 flow of 80 sccm for approximately 2 minutes.

Section 4. Propagation of our vortex beam in free space

For a better understanding of the unique intensity profiles in our vortex beams, we simulated the free-space propagation of our vortex beam across the Fresnel and Fraunhofer regions. In our simulations, assuming that a normally incident plane wave has the electric field $U(x,y,z=0)$, we can get the diffracted field bed by angular spectrum method

$$u(x,y,z)=\iint_{\infty}\hat{A}(\kappa_x,\kappa_y)\cdot e^{i(k_x x+k_y y+k_z z)}dk_x dk_y \tag{S5}$$

where

$$\hat{A}(k_x,k_y)=\frac{1}{4\pi^2}\iint_{\infty}U(x,y,0)\cdot g(x,y)\cdot e^{-i(k_x x+k_y y)}dxdy \tag{S6}$$

where $\hat{A}(k_x,k_y)$ is the Fourier transform of electric field, $g(x,y)=T(x,y)\cdot circ(r_d/d_q)$, $r_d^2=x^2+(y-d_q/2)^2$, *circ* is the circular function describing the aperture and k_x, k_y *and* k_z denote respective components of wave vector k.

The simulated results in Fig. S4 help us to recognize the physical evolution of our vortex beam from a uniform illumination with a spiniform wavefront to the unique intensity profiles. Due to the linear phase dislocation along y axis as shown in Fig. S1(c), the dark region in the diffraction pattern near the vortex generator has a y-direction line shape around every phase singularity. Since the spiniform phase is taken as the superposition of helical phase and has no propagation in-variation, its corresponding phase and intensity profile will have a rapid change in the Fresnel region. Figure S4 gives the detailed intensity profiles including the initial, rapidly changed (Fresnel region) and stable patterns (Fraunhofer region). For q=1, only one singularity is located at the center of diffracting beam so that the resulting pattern in Fraunhofer region maintains a doughnut shape, which is similar to LG beam. However, when q≥2, there will be multiple spatially-discrete phase singularities along y aixs, which will change their relative locations during the propagation in the Fresnel region. For the light without phase singularities in positive and negative-x region, they will propagate with the confinement of circular aperture whose diffraction pattern has the outmost ring with larger ring-width and many inner rings with gradually decreased ring-width, which can be found at z=0.2mm for L=1 in Fig. S4. In the Fraunhofer region, these two regions without phase singularities will lead to two bright lobes with their relative phase singularities located between. When q is larger, the dark region dominated by more singularities will extend, which will increase the distance between two lobes. This is the reason why our vortex beam has two-lobe intensity profile for q≥2 and the distance between two lobe enlarges with the increment of q. Shortly, it is the complex interaction between spiniform phase and circular aperture.

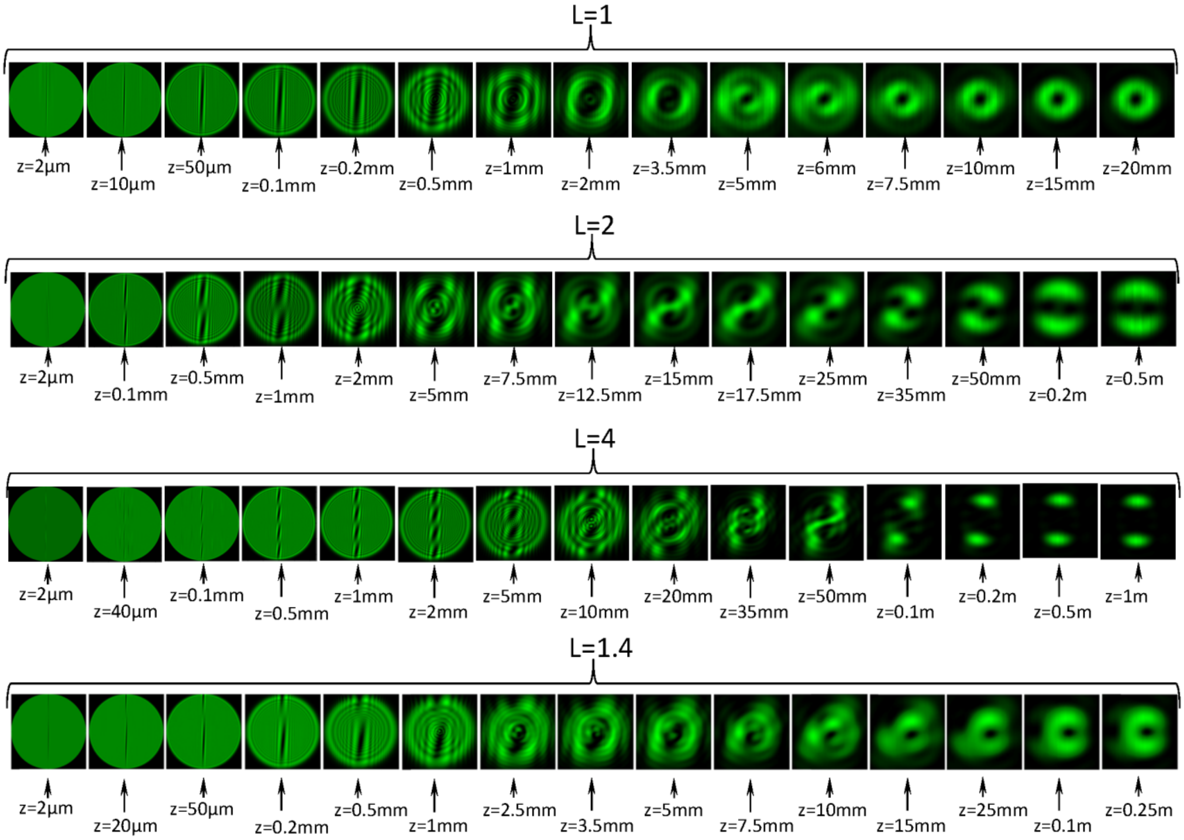

Figure S4. Free-space propagation of our vortex beam with *q*= 1, 2, 4 and 1.4. Their parameters of all the cases are the same with those in Fig. 2 and 3.

Section 5. Experiment for charactering the vortex beam

In order to reveal the phase structure of the generated vortex beam by our transmitter, we have built up an experimental setup for measuring its interference with a plane and spherical wave via Mach-Zehnder interferometer, as shown in Fig. S5. Light from a laser is coupled into a mono-mode fibre and then collimated by an objective lens L_1 with its magnification of 5×. The collimated light with its diameter of about 11mm is divided into two beams by a 50-50 beam splitter (BS). One beam as a main beam is used to illuminate the vortex generator with the diameter at the magnitude order of hundreds of micro-meters so as to obtain the uniform illumination. Its transmitted light will form a slightly divergent optical vortex beam, which is collimated by a lens L_2 for a better interference pattern, especially for the case of the reference beam with a plane wave. The collimated vortex beam is reflected by a mirror M_1 for its normal incidence on another beam-splitter BS_2. The vortex beam passing through BS_2 is taken as the main beam for interfering with the reference beam.

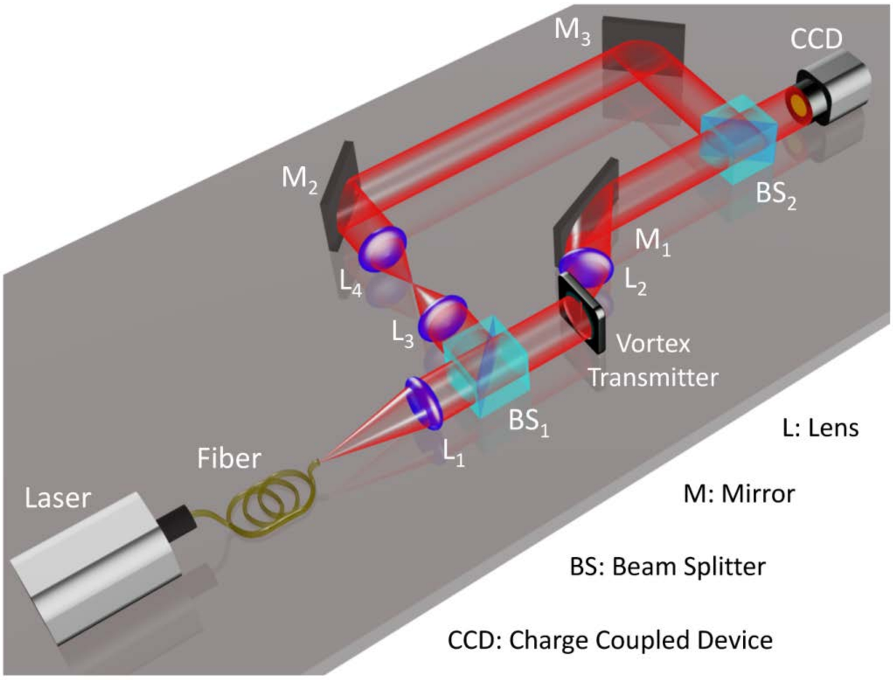

Figure S5. Mach-Zehnder interferometer for measuring the phase profile.

The other beam splitted from BS_1 is used as the reference beam. This beam is manipulated by two lenses (L_3 and L_4) with the same focal length of 50mm. In order to tune the wavefront (plane wave or spherical wave) of reference beam in experiment, we fix the lens L_3 and move the lens L_4 along the propagating direction of light. When these two lenses are confocal at a same position, light after L_4 is a collimated beam as a plane wave. Then, we move the lens L_4 forward (backward) along the propagating direction of light, resulting that a focused (divergent) spherical wave is generate. After the reflection precisely tuned by two mirrors (M_2 and M_3), the reference beam is normally incident on the 50-50 BS_2. One part of reference beam from BS_2 co-propagates with the main beam in a slight tilting angle so as to observe the interference pattern by a charge-coupled device (CCD).

If the reference beam is tuned for a plane wave, the interference pattern captured by the CCD is the dislocated fringes as shown in Fig. S6 (a), which is in good agreement with simulated results in Fig. S6(b). The direction of fringes in the interference pattern can be changed by slightly tuning the mirrors (M_2 and M_3). Also, the width of fringes can be tuned by changing the angle between the reference and main beam with the help of the mirror M_3. The wide fringe means a small angle.

If the reference beam has a wavefront of a spherical wave, its interference pattern has the spiral pattern with different arms of q for various vortex beams, which is shown in Fig. S6(c). The good consistency between simulation and experiment confirms the validation of our method.

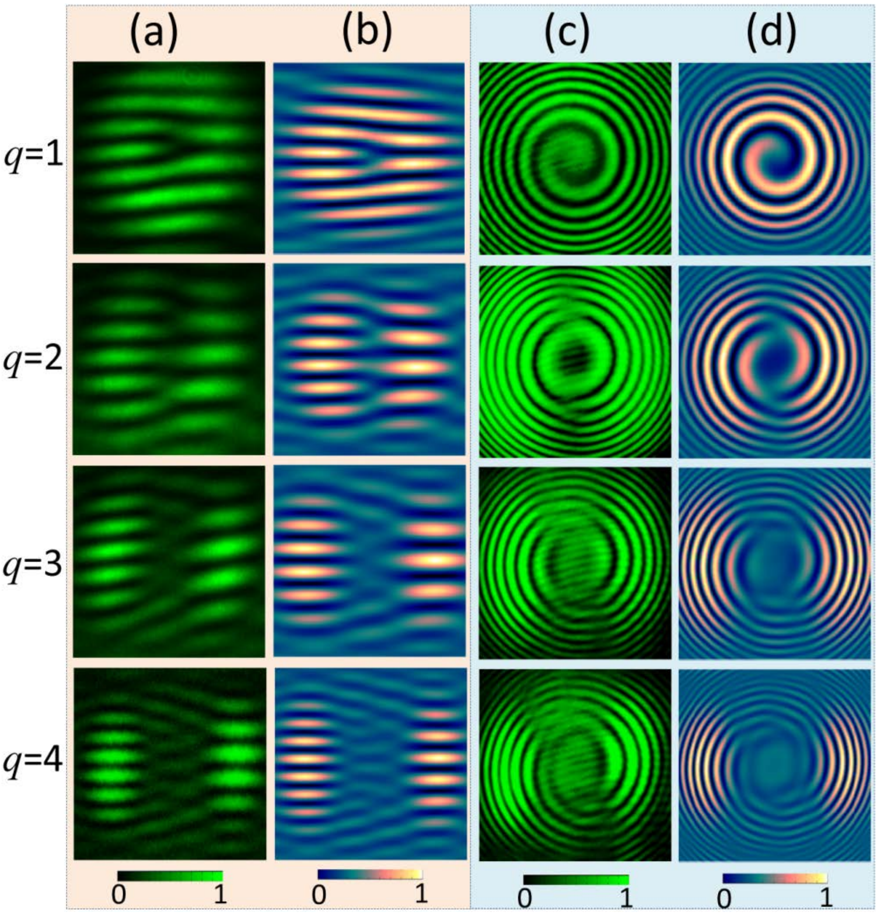

Figure S6. Interference patterns. Experimental (a) and simulated (b) interference patterns between our vortex beam and a plane wave. Experimental (c) and simulated (d) interference patterns between our vortex beam and a spherical wave.

Section 6. Phase retrieval from experimental interference

To obtain the phase from experimental interference, we use the signal processing technique with the cropping of frequency spectrum on the basis of Fourier transformation. Ignoring its principle theory (one can refer to Ref. [S5]) for this technique, we only introduce several key steps as shown in Fig. S7(a):

Step 1: Fourier transformation of experimental interference. Fourier transformation is realized by fast Fourier transformation (FFT) that shows a good approximation. In addition, experimental interference pattern with dense fringes is preferred for a better retrieval because this vortex beam for this case is encoded into high-frequency information that is apparently separated from the low-frequency, leading to an easier operation in selecting and cropping its frequency spectrum. In the diagram for frequency spectrum of Fig. S7(a), the top and bottom white rectangles respectively denote

the frequency information of ±q vortex beam while the green rectangle stand for that of reference beam. In practice, we only need to retrieve the information of one beam such as +q vortex.

Step 2: Selecting the useful frequency spectrum (denoted by white dashed rectangle), removing the other frequency spectrum, shifting the useful frequency spectrum to the centre and generating the new frequency spectrum for final retrieval. The detailed operations are sketched in Fig. S7.

Step 3: Inverse Fourier transformation of cropped frequency spectrum. Correspondingly, the inverse FFT is employed to carry out the final retrieval from the cropped frequency spectrum. Thus, we can get the retrieved amplitude and phase profiles from the experimental interference, as shown in Fig. S7(a).

The above retrieval steps are only suitable for the fringed pattern interfered with a plane wave because the frequency domain of vortex beam is separated from the low frequency carried by the reference beam. For the spherical-wave cases, it is difficult by using this technique because the frequency information of vortex beam and reference beam overlays with each other.

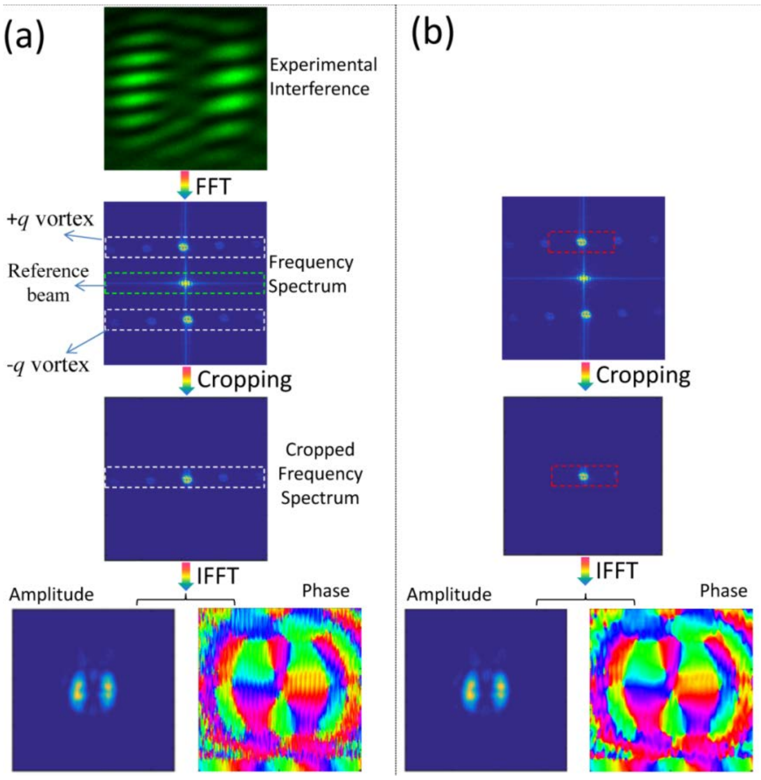

Figure S7. Phase retrieval from experimental interference. (a) Steps in carrying out the phase retrieval. It contains three steps: 1) Fourier transformation of experimental interference; 2) Cropping the useful frequency spectrum and shifting it to the centre; 3) Inverse Fourier transformation of cropped frequency spectrum. The case for q=3 vortex beam is taken as an example. **(b)** Phase retrieved from a small-sized cropped frequency spectrum without high frequency. This allows us to get a smoother phase pattern.

Another important issue is that, the size of selected frequency area has an obvious influence on the final retrieved results, especially for the smoothness of phase profile. To show this, we select a small sized frequency area for retrieval, as depicted in Fig. S7(b), keeping other operations the same with those in Fig. S7(a). Its smoothness of retrieved phase is better than that of the large-sized case while the amplitude has no obvious change, which shows that the phase of light is more sensitive than amplitude.

Section 7. Calculating orbital angular momentum carried by our vortex beam

Considering the fact that our optical vortex beam is paraxial, we use the theory by Allen et al. to calculate its OAM [S6]. Because of the non-axisymmetry of our vortex beam, we evaluate its OAM by using the version in Cartesian coordinates.

For a vector potential $\boldsymbol{A}$ polarized along $\boldsymbol{x}$,

$$\mathbf{A} = \mathbf{e}_\mathbf{x} \cdot u(x, y, z)\exp(ikz), \tag{S7}$$

the paraxial approximation, after use of the Lorentz gauge for convenience [S7], yields:

$$\mathbf{B} = \mu_0 \mathbf{H} = \nabla \times \mathbf{A} = \begin{bmatrix} \mathbf{e}_x & \mathbf{e}_y & \mathbf{e}_z \\ \frac{\partial}{\partial x} & \frac{\partial}{\partial y} & \frac{\partial}{\partial z} \\ u \cdot e^{ikz} & 0 & 0 \end{bmatrix} = \left(\frac{\partial u}{\partial z} e^{ikz} + ikue^{ikz} \right) \mathbf{e}_y - \frac{\partial u}{\partial y} e^{ikz} \mathbf{e}_z$$
$$\approx ike^{ikz} \left(u\mathbf{e}_y + \frac{i}{k} \frac{\partial u}{\partial y} \mathbf{e}_z \right) \tag{S8}$$

$$\mathbf{E} = -i\omega\mathbf{A} - \nabla \cdot \left(\frac{i}{\omega\varepsilon_0\mu_0} \nabla \cdot \mathbf{A} \right) = -i\omega ue^{ikz}\mathbf{e}_x - \nabla \cdot \left(\frac{ic^2}{\omega} \frac{\partial u}{\partial x} e^{ikz} \right)$$
$$= -i\omega ue^{ikz}\mathbf{e}_x - \frac{ic^2}{\omega} \left[\frac{\partial^2 u}{\partial x^2} e^{ikz}\mathbf{e}_x + \frac{\partial^2 u}{\partial x \partial y} e^{ikz}\mathbf{e}_y + \left(\frac{\partial^2 u}{\partial x \partial z} e^{ikz} + ik\frac{\partial u}{\partial x} e^{ikz} \right)\mathbf{e}_z \right]$$
$$= \omega \left\{ -iue^{ikz}\mathbf{e}_x - \frac{ic^2}{\omega^2} \left[\frac{\partial^2 u}{\partial x^2} e^{ikz}\mathbf{e}_x + \frac{\partial^2 u}{\partial x \partial y} e^{ikz}\mathbf{e}_y + \left(\frac{\partial^2 u}{\partial x \partial z} e^{ikz} + ik\frac{\partial u}{\partial x} e^{ikz} \right)\mathbf{e}_z \right] \right\} \tag{S9}$$
$$\approx \omega \left\{ -iue^{ikz}\mathbf{e}_x - \frac{i}{k^2} \left[ik\frac{\partial u}{\partial x} e^{ikz}\mathbf{e}_z \right] \right\} = -i\omega \left(u\mathbf{e}_x + \frac{i}{k}\frac{\partial u}{\partial x}\mathbf{e}_z \right) e^{ikz}$$

Then, we have some basic formulas about the cross product of **E** and **B**:

$$\mathbf{E}^* \times \mathbf{B} = \begin{bmatrix} \mathbf{e}_x & \mathbf{e}_y & \mathbf{e}_z \\ i\omega u^* & 0 & \frac{\omega}{k}\frac{\partial u^*}{\partial x} \\ 0 & iku & -\frac{\partial u}{\partial y} \end{bmatrix} = \left(-i\omega u \frac{\partial u^*}{\partial x}\mathbf{e}_x + i\omega u^* \frac{\partial u}{\partial y}\mathbf{e}_y + k\omega |u|^2 \mathbf{e}_z \right), \tag{S10}$$

$$\mathbf{E} \times \mathbf{B}^* = \begin{bmatrix} \mathbf{e}_x & \mathbf{e}_y & \mathbf{e}_z \\ -i\omega u & 0 & \frac{\omega}{k}\frac{\partial u}{\partial x} \\ 0 & -iku^* & -\frac{\partial u^*}{\partial y} \end{bmatrix} = \left(i\omega u^* \frac{\partial u}{\partial x}\mathbf{e}_x - \omega u \frac{\partial u^*}{\partial y}\mathbf{e}_y + k\omega |u|^2 \mathbf{e}_z \right), \tag{S11}$$

Using Eqs. (S10) and (S11), we get the linear momentum density as follows:

$$\begin{aligned} \varepsilon_0 \langle \mathbf{E} \times \mathbf{B} \rangle &= \frac{\varepsilon_0}{2}\left[(\mathbf{E} \times \mathbf{B}^*) + (\mathbf{E}^* \times \mathbf{B}) \right] \\ &= \frac{\varepsilon_0}{2}\left(i\omega u^* \frac{\partial u}{\partial x}\mathbf{e}_x - \omega u \frac{\partial u^*}{\partial y}\mathbf{e}_y + k\omega |u|^2 \mathbf{e}_z - i\omega u \frac{\partial u^*}{\partial x}\mathbf{e}_x + i\omega u^* \frac{\partial u}{\partial y}\mathbf{e}_y + k\omega |u|^2 \mathbf{e}_z \right), \\ &= \frac{i\varepsilon_0\omega}{2}\left[\left(u^* \frac{\partial u}{\partial x} - u \frac{\partial u^*}{\partial x} \right)\mathbf{e}_x + \left(u^* \frac{\partial u}{\partial y} - u \frac{\partial u^*}{\partial y} \right)\mathbf{e}_y \right] + \varepsilon_0 k\omega |u|^2 \mathbf{e}_z \end{aligned} \tag{S12}$$

$$\begin{aligned} \varepsilon_0 (\mathbf{r} \times \langle \mathbf{E} \times \mathbf{B} \rangle) &= [x, y, z] \times \left\{ \frac{i\varepsilon_0\omega}{2}\left[\left(u^* \frac{\partial u}{\partial x} - u \frac{\partial u^*}{\partial x} \right)\mathbf{e}_x + \left(u^* \frac{\partial u}{\partial y} - u \frac{\partial u^*}{\partial y} \right)\mathbf{e}_y \right] + \varepsilon_0 k\omega |u|^2 \mathbf{e}_z \right\} \\ &= \left[y\varepsilon_0 k\omega |u|^2 - z\frac{i\varepsilon_0\omega}{2}\left(u^* \frac{\partial u}{\partial y} - u \frac{\partial u^*}{\partial y} \right) \right]\mathbf{e}_x + \left[-x\varepsilon_0 k\omega |u|^2 + z\frac{i\varepsilon_0\omega}{2}\left(u^* \frac{\partial u}{\partial x} - u \frac{\partial u^*}{\partial x} \right) \right]\mathbf{e}_y , \\ &\quad + \frac{i\varepsilon_0\omega}{2}\left[x\left(u^* \frac{\partial u}{\partial y} - u \frac{\partial u^*}{\partial y} \right) - y\left(u^* \frac{\partial u}{\partial x} - u \frac{\partial u^*}{\partial x} \right) \right]\mathbf{e}_z \end{aligned} \tag{S13}$$

Therefore, according to Allen's definition, the ratio of angular momentum to energy per unit length of beam can be expressed as

$$\frac{J_z}{W} = \frac{\varepsilon_0 \iint dxdy (\mathbf{r} \times \langle \mathbf{E} \times \mathbf{B} \rangle)_z}{c\varepsilon_0 \iint dxdy (\langle \mathbf{E} \times \mathbf{B} \rangle)_z} = \frac{\iint M_z dxdy}{\iint j_z dxdy}, \tag{S14}$$

where

$$M_z = \frac{i\varepsilon_0\omega}{2}\left[x\left(u^* \frac{\partial u}{\partial y} - u \frac{\partial u^*}{\partial y} \right) - y\left(u^* \frac{\partial u}{\partial x} - u \frac{\partial u^*}{\partial x} \right) \right], \tag{S15}$$

$$j_z = c\varepsilon_0 k\omega |u|^2, \tag{S16}$$

Finally, we have the ratio

$$\frac{J_z}{W} = \frac{i}{2\omega}\frac{\iint \left[x\left(u^* \frac{\partial u}{\partial y} - u\frac{\partial u^*}{\partial y}\right) - y\left(u^* \frac{\partial u}{\partial x} - u\frac{\partial u^*}{\partial x}\right)\right] dxdy}{\iint |u|^2 dxdy}, \tag{S17}$$

which is usually used to evaluate the average OAM (in unit of $\hbar$) of photon in unit length.

By using Eq. (S17), we can calculate the average OAM carried by our vortex beam once its electric field with amplitude and phase is given. In simulation, the electric field can be easily obtained by employing Eq. (S5). It is worthy to point out that the calculation axis in our simulation is the symmetrical axis (perpendicular to the aperture plane and through the original point) of the circular aperture so as to remove the additional extrinsic OAM due to the mismatch between the calculation axis and symmetrical axis of optical system at the entrance plane [S8]. However, its electric field in experiment can only be retrieved from its experimental interference, which has been described above. All the simulated and experimental results about its average OAM are provided in Fig. 1(f) of main text. In addition, we also give a curve fitting of simulation results with a root mean square error of 0.04 and find that its OAM per photon has an analytical dependence on q: Q=0.7q-sin(πq/2)•sinc(πq/2)/2, which shows a clear description about its average OAM value and also finally confirms the analog generation of rational OAM by using our proposal.

For Laguerre-Gaussian and Bessel beams having a helical phase of exp($il\varphi$) (where l is the topological charge and φ is the angle coordinate), their carried OAMs (in units of $\hbar$) have the form of L=l-sin(2$l\pi$)/(2π) [S9-S10], showing a non-linearity dependence on its topological charge l. For integer l, they have a well-defined OAM of $l\hbar$. But for fractional l, sin(2$l\pi$)/(2π) works as a modification item for its carried OAM. This is mainly because the phase exp($il\varphi$) for fractional l can be taken as a weighted super-position of exp($il\varphi$) with integer l. In addition, optical vortex beams with fractional l have the non-axisymmetric intensity profiles, which are induced by the discontinuity of helical phase in angular position. As a result, when l varies from an integer to its neighbouring integer, LG and Bessel beams have their intensity profiles changing from axisymmetry, to non-axisymmetry and axisymmetry, showing a regular variation tendency.

However, for our vortex beam, there is no such obvious tendency. For the case 0<q<1, its phase and intensity have a similar behaviour (from a plane wave for q=0 to a vortex beam with a ring intensity for q=1) with that of LG and Bessel beam so that the *sine* item of our case dominates this region. Then, when q increases from 1 to 2, its intensity profile changes from a ring shape to two-lobe shape, showing a non-linear variation. This physically explain the non-linear dependence of Q on q in the range from q=0 to q=2 in Fig. 1(f). For q>2, the vortex beam keeps a two-lobe intensity, having a linear variation that is dominated by the *sinc* item. Correspondingly, the item sin(πq/2)•sinc(πq/2)/2 tends to be zero for q>2, leaving a linear item of 0.7q .

Section 8. Quantum operations in simulation and experiment

8.1. Spiral spectrum of a super-position state of OAM

Mathematically, a 2-dimensional function can be expressed by a weighted superposition of the spiral harmonics. This mathematical concept has been introduced into the physics regime for spiral imaging by using the measured weighted coefficients, i.e., the spiral spectrum. Here, we give a brief introduction about the theory of spiral imaging, which was built by Toner, Torres and Carrasco [S11]. We here follow

their steps to introduce the spiral imaging theory. For a field distribution with $u(\rho,\varphi)$, one can obtain its spiral spectrum by projecting it into the spiral harmonics $\exp(in\varphi)$

$$u(\rho, \varphi) = \frac{1}{\sqrt{2\pi}} \sum_{n=-\infty}^{\infty} \lambda_n(\rho) \exp(in\varphi), \tag{S18}$$

where the weighted function

$$\lambda_n = \frac{1}{\sqrt{2\pi}} \int_0^{2\pi} u(\rho, \varphi) \exp(-in\varphi) d\varphi. \tag{S19}$$

One can find that the field distribution $u(\rho,\varphi)$ can be reconstructed if we know $a_n(\rho)$ for every spiral harmonics. But, it is very difficult to get $a_n(\rho)$ through the direct experimental measurement. The variable that can be measured in experiment is the spiral spectrum, which is defined as

$$P_n = \frac{1}{T} \int_0^{\infty} |\lambda_n(\rho)|^2 \rho d\rho, \tag{S20}$$

where the normalized constant $T = \sum_{n=-\infty}^{\infty} P_n$.

Two effective measurement methods of measuring the spiral spectrum are: 1) traditional mode decomposition based on the analysis hologram and optical low-pass filtering system [S12]; 2) entanglement measurement based on spontaneous parametric down-conversion [S13].

8.2. Simulation about the spiral spectra of our vortex beams

To simulate the probability amplitude λ_n of a spiral mode contained in our vortex beams, we use the method reported in Ref. S12. Light with our spiniform phase passes through a helical phase of $\exp(-in\varphi)$ as a probe of n-order spiral mode and then is focused by a thin lens with a focal length of f. The electric field at the focal plane can be described as [S14]

$$E(x,y,f) = A \iint_{-\infty}^{\infty} \chi(x_0,y_0) \cdot circ(r_d/d_q) \cdot exp(-in\varphi) exp\left(-i\frac{2\pi(x_0x+y_0y)}{\lambda f}\right) dx_0 dy_0, \tag{S21}$$

where A is a function independent on x_0 and y_0 and can be taken as a constant here. Since our vortex beam origins from the encircled spiniform phase of $\chi(x_0,y_0) \cdot circ(r_d/d_q)$, both have the same spiral spectrum so that we use $\chi(x_0,y_0)$ instead of $u(x_0,y_0)$ in Eq. (S21) for an easy operation. One can easily find that $\lambda_n \propto E(0,0,f)$, which can be calculated directly by employing a fast Fourier transformation of $(x_0,y_0) \cdot circ(r_d/d_q) \cdot exp(-in\varphi)$ or carrying out the optical propagation of a lens-based focusing system [S15]. We have to emphasize that Eq. (S21) is a quite efficient method to analyze the spiral spectrum of any beam (i.e., not limited to a vortex beam).

8.3. Experimental measurement about quantum entanglement

The single-photon experiment is carried out on the basis of SPDC in Fig. 4a of main text. The correlated single-photon pairs are generated by using a type-1 3mm-long β-barium-borate (β-BBO) crystal pumped by a collimated CW laser at $\lambda_{2\omega}$ = 355 nm. After being bumped by a UV laser, the single-photon source with 710nm wavelength has a transverse mode of a Gaussian beam with a diameter of 1mm, which is enlarged to 2mm by a beam expander composed of lenses L_1 and L_2. The idler and signal fields are then separated by a beam splitter and imaged onto two individual SLMs (see Fig. 4a in the main text). In both arms, SLM_1 and SLM_2 are employed to select the vortex beams by encoding the corresponding phase profiles. To generate our vortex beam conveniently for easy experimental operation, we change $q_A=d_q/\tau$ in the signal arm by tuning the parameter τ and keeping d_q=0.6mm, which is a little different from Fig. 1 but still able to demonstrate the quasi-continuous generation of our vortex beam due to the smooth variation of q_A, see Fig. S8 for more details. Therefore, such an alternative

approach will not change any properties of our vortex beam and is able to verify the feasibility of our vortex beams in carrying out quantum operation. For this case, the origin of coordinate for the phase profile in Fig. 1c is located at the bottom of the circular aperture on the SLM, see Fig. 4b in the main text.

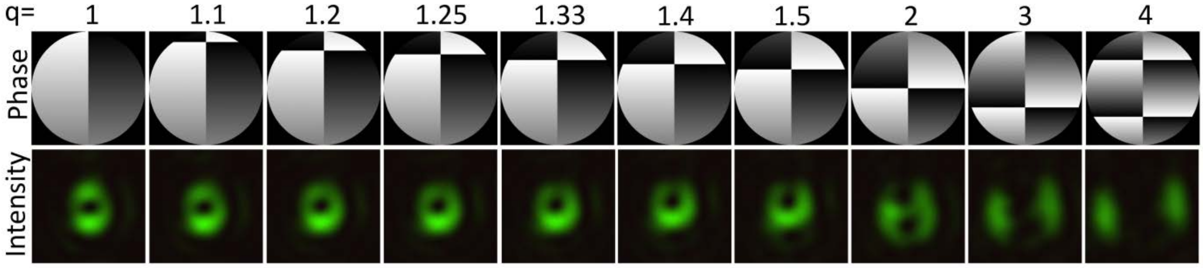

Figure S8. The vortex beams generated by changing τ with a fixed aperture of 4mm in diameter. The operating wavelength is chosen to be 532nm so that a fair comparison is achieved under the same condition. The upper and lower rows show the encoded phase profiles and their measured intensity at the far field. It shows that the far-field intensity patterns are the same with those in maix text, which verifies the feasibility of imitating the generation of our vortex beams by using SLM.

To scan all the interested vortex beams for the measurement of quantum spiral spectrum and coincidence, Labview software is employed to control both SLMs through writing the required phase to the SLMs sequentially. The experimental coincidence counts in 4 seconds are provided in Fig. 4c and 4d in the main text.

It is important to appreciate that there are various constraints on the experimental system which require tradeoffs to be made between the signal strength, effective pinhole diameter and quantum spiral bandwidth. The maximum bandwidth is obtained through a combination of large pump beam and thin crystal, but an extremely thin crystal would result in a too-low optical flux [S16]. Similarly the area illuminated by the pump beam itself needs to be magnified to utilize the aperture of the spatial light modulator and the spinform phase and then demagnified to the facet of single mode fibre core, all with high optical efficiency. Finally, the measurement fibre aperture itself sets the limiting aperture of the experiment because the aperture is effectively Gaussian [S17] in profile (*i.e.* not top-hat) due to the single-mode nature of the coupling. To better approximate a flat topped beam compared to a Gaussian beam, a 0.6 mm-diameter pinhole (see Fig. 4b in the main text) can be added onto SLM and will act as an aperture on the Gaussian mode detected by the fibre albeit at the expense of detection efficiency. The final experimental configuration is an optimized compromise between the competing factors of flux and spiral bandwidth.

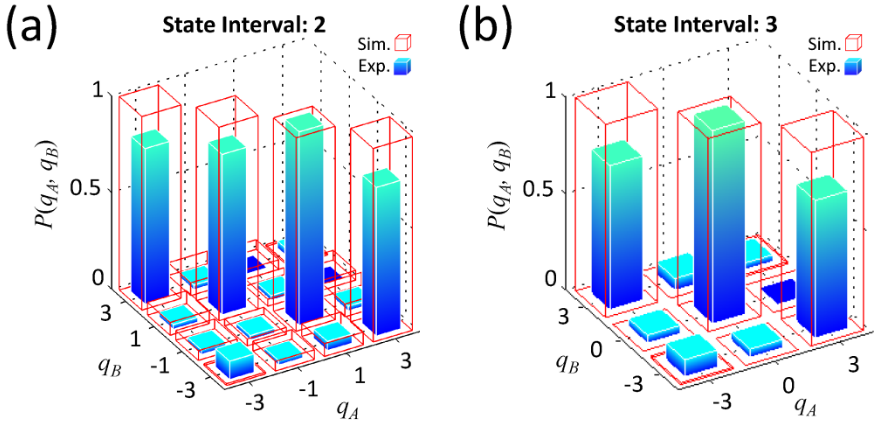

Figure S9. Quantum coincidence between discrete states with the intervals of 2 (a) and 3 (b).

8.4. Decreasing the crosstalk in quantum coincidence by using larger state intervals

Considering that our vortex beams have non-integer OAMs in terms of OAM-superposition states, we use the method of increasing the state interval to suppress the crosstalk in quantum coincidence between our vortex beam with discrete q_A and q_B. Limited by our experimental configuration, we captured the coincidence rates in the range of $q_{A,B}$ ϵ[-3, 3]. The following discussions are based on the existing experimental results. For the case with the state interval of 2, we used the state space of $q_{A,B}$ =±1 and ±3. The corresponding experimental and simulated coincidences are shown in Supplementary Figure S9. The crosstalks are -11.81 in simulation and -10.24 in experiment, which are lowered a little compared with the case of interval 1. When the state interval equals 3, the simulated and expriemntal coincidences with $q_{A,B}$ =0 and ±3 are shown in Supplementary Figure S9(b). The simulated crosstalk is suppressed down to -20.36 although the experimental crosstalk of -10.56 has no significant decrement due to the experimental error caused by the imperfection of experimental vortex beam and strong noise. It is believed that the ultra-low crosstalk could be achieved after a detailed optimization of the experimental measurement system. Physically, such a method of increasing state interval is employed here because the overlapping between two neighbouring states could be avoided, which is similar to the orthogonality of pure-OAM basis. In real applications, our proposed bilateral grating with a tunable aperture is quite efficient in selecting the required vortex beams, which paves a solid path to popularize our technique for communication purpose.

References:

S1. J. B. Gotte, S. Franke-Arnold, R. Zambrini, and S. M. Barnett, "Quantum formulation of fractional orbital angular momentum", *J. Mod. Opt.* **54**, 1723-1738(2007).

S2. R. Rickler, R. Lapkiewicz, W. N. Plick, M. Krenn, C. Schaeff, S. Ramelow, and A, Zeilinger, "Quantum entanglement of high angular momenta", *Science* **338**, 640-643 (2012).

S3. J. W. Goodman, Introduction to Fourier Optics, (Roberts & Company Publishers, 2005).

S4. I.V. Basistiy, V. Y. Bazhenov, M. S. Soskin, and M. V. Vasnetsov, "Optics of light beams with screw dislocations," *Opt. Comm.* **103**, 422-428 (1993).

S5. M. Takeda, H. Ina, and S. Kobayashi, "Fourier-transform method of fringe-pattern analysis for computer-based topography and interferometry," *J. Opt. Soc. Am.* **72**, 156-160 (1982).

S6. L. Allen, M. W. Beijersbergen, R. J. C. Spreeuw, and J. P. Woerdman, "Orbital angular-momentum of light and the transformation of Laguerre–Gaussian laser modes," *Phys. Rev. A* **45**, 8185–8189 (1992).

S7. H. A. Huas, 'Waves and fields in optoelectronis'. (Prentice-Hall Englewood Cliffs, 1984).

S8. A. T. O'Neil, I. Mac Vicar, L. Allen, and M. J. Padgett, "Intrinsic and extrinsic nature of the orbital angular momentum of a light beam", *Phys. Rev. Lett.* 88, 053601(2002).

S9. J. Leach, E. Yao, and M. J. Padgett, "Observation of the vortex structure of a non-integer vortex beam," *New J. Phys*. **6**, 71 (2004).

S10. J. C. Gutierrez-Vega, and C. Lopez-Mariscal, "Nondiffracting vortex beams with continuous orbital angular momentum order dependence," *J. Opt. A: Pure Appl. Opt*. **10**, 015009 (2008).

S11. L. Torner, J. P. Torres, and S. Carrasco, "Digital spiral imaging", Opt. Express 13, 873-881(2005).

S12. Franke-Arnold, et al., “Uncertainty principle for angular position and angular momentum” New Journal of Physics 6, 103(2004).

S13. L. Chen, J. Lei, and J. Romero, “Quantum digital spiral imaging”, Light Sci. Appl. 3, e153(2014).

S14. J.W. Goodman, Introduction to Fourier Optics, Roberts & Company Publishers, 2005.

S15. K. Huang, et al., “Design of diffractive phase element for modulating the electric field at the out-of-focus plane in a lens system”, *Appl. Opt.* **51**, 5149-5153(2012).

S16. J. P. Torres, A. Alexandrescu, L. Torner, “Quantum spiral bandwidth of entangled two-photon states”, *Phys. Rev. A* **68**, 050301(R) (2003).

S17. S. Restuccia, D. Giovannini, G. Gibson, M. Padgett, “Comparing the information capacity of Laguerre-Gaussian and Hermit-Gaussian modal sets in a finit-aperture system”, *Opt. Express* **24**, 27127-27136(2016).
